# Socio-economic, infrastructural and health-related risk factors associated with adverse heat-health effects reportedly experienced during hot weather in South Africa

**DOI:** 10.11604/pamj.2019.34.40.17569

**Published:** 2019-09-18

**Authors:** Caradee Yael Wright, Friederike Dominick, Thandi Kapwata, Shalin Bidassey-Manilal, Jacobus Christoffel Engelbrecht, Heribert Stich, Angela Mathee, Mamopeli Matooane

**Affiliations:** 1 Environment and Health Research Unit, South African Medical Research Council, Pretoria, South Africa; 2 Department of Geography, Geoinformatics and Meteorology, University of Pretoria, Pretoria, South Africa; 3 Ludwig-Maximilians-Universität, München, Germany; 4 Environment and Health Research Unit, South African Medical Research Council, Johannesburg, South Africa; 5 University of Johannesburg and Department of Environmental Health, Tshwane University of Technology, Johannesburg, South Africa; 6 Department of Environmental Health, Tshwane University of Technology, Pretoria, South Africa; 7 Health Department, Landshut, Germany; 8 University of Johannesburg, Johannesburg, South Africa; 9 University of the Witwatersrand, Johannesburg, South Africa; 10 Tlhoeko Environmental Consultants, Maseru, Lesotho; 11 Previously Natural Resources and the Environment Unit, Council for Scientific and Industrial Research, Pretoria, South Africa

**Keywords:** Climate change, environmental health, heat, temperature exposure, South Africa

## Abstract

**Introduction:**

Poor urban communities are likely to bear the brunt of climate change impacts on health and well-being. The City of Johannesburg, South Africa, is predicted to experience an average increase in ambient temperature of 4°C by 2100. Focusing on the urban environment, this study aimed to determine socio-economic, infrastructural and health-related risk factors for heat-related adverse health effects.

**Methods:**

This was a cross-sectional study. Data of interest were collected using a pretested and validated questionnaire administered to parents of children attending schools participating in a school heat study. Information related to demographic, socio-economic and household-level determinants of health, which has an impact on the individual prevalence of adverse heat-health effects associated with hot weather, was collected for 136 households and 580 individuals.

**Results:**

Sweating (n = 208 individuals; 35%), headache and nausea (n = 111; 19%) and weakness, fatigue and dizziness (n = 87; 15%) were the most common heat-health effects reportedly experienced by individuals (n = 580) during hot weather. Individuals who suffered from hypertension (OR = 2.32, 95% CI: 1.34 - 4.05, p = 0.003) and individuals older than 60 years (OR = 1.81, 95% CI: 1.27-1.99, p < 0.001) compared to other age groups were more likely to experience 'any heat-health effects'. Living in government-sponsored detached housing and in houses with asbestos roofs were associated with an increase in reported experience of 'any heat-health effects' compared to living in other housing types.

**Conclusion:**

Heat-health awareness campaigns should target people suffering from pre-existing diseases and the elderly, as these groups are especially vulnerable to heat. Focus should also be given to appropriate roofing and insulation in government-sponsored housing since summertime temperatures are projected to increase.

## Introduction

In a changing climate, many parts of the world are experiencing steadily increasing temperatures [1]. While much uncertainty about climate change remains, the few studies of large, low-and middle-income country populations (specifically in the tropics) suggest possible effects of heat, and less so effects of cold, on mortality [2]. In some parts of the world, climate change is exacerbating health problems by increasing risks of morbidity and mortality caused by intense heat episodes [3]. Those groups most vulnerable to these impacts tend to be indigent communities living in high-density urban settings [4], as well as children [5], the elderly [6], people with disabilities [7] and pre-existing diseases [4] and families from lower socio-economic groups [8]. Africa is projected to see significant increases in the number of days when health may be adversely affected by increasing maximum temperatures due to climate change [9]. These increases in Africa may occur at a rate faster than elsewhere [10, 11]. The ability of people to acclimatise to relatively rapid and sustained change in temperature, such as those predicted for climate change, is mostly unknown [12, 13]. People are likely to experience heat-related health impacts including discomfort, fatigue and heat stroke [14]. The association between high temperature and increased mortality from all-cause mortality, as well as cardiovascular, respiratory and cerebrovascular diseases, has been demonstrated internationally and in parts of Africa [15-17]. In northern Ghana, for example, all-cause mortality was statistically significantly associated with mean daily temperatures above the 75th percentile (the threshold applied in that study) [18]. Very little is known about heat-related morbidity in Africa. Some work to understand possible heat-health symptoms and impacts has been done in South Africa. Indoor temperatures measured in waiting rooms in rural clinics indicated that maximum daily temperatures exceeded 38°C [19]. The difference between indoor and outdoor temperatures was between 2-4°C on average with warmer termperatures experienced in the clinic waiting rooms. 'Real-feel' temperatures, that include humidity measurements, were 4°C higher than measured indoor temperature. This suggests that patients waiting for treament may have experienced a feeling of 'stuffiness' and discomfort [20]. A study conducted in the City of Johannesburg (CoJ) found that students (n = 252) aged ~14-18 years felt tired and found it hard to breathe when classroom temperatures ('real-feel') were greater than 32°C [21]. Higher indoor classroom temperatures were consistently observed in classrooms constructed of prefabricated asbestos sheeting with corrugated iron roofs and converted shipping containers, compared to brick and other materials used in classroom construction. In South Africa, about 11% of the urban population live in informal settlements [22] where levels of poverty and overcrowding are usually high, and housing comprises mainly shacks constructed using metal and plastic sheeting. Other settings of urban poverty may comprise areas of inner-city degradation and government-built brick dwellings with basic amenities (but not necessarily ceilings). These residents are extremely vulnerable to variations in ambient temperature. Insight is needed into heat-health effects and associated risk factors, including those related to dwelling characteristics, to help plan for public health interventions to reduce heat-related morbidity and mortality during high temperatures and extreme heat events in Africa. Therefore, this study aimed to determine socio-economic, infrastructural and health-related risk factors associated with heat-health effects reportedly experienced during hot weather in an African urban environment.

## Methods

**Study site and data collection:** this was a cross sectional study. Data of interest were collected using a pretested and validated questionnaire in the CoJ. The CoJ, located in Gauteng province, South Africa, has on average daily maximum temperature ranges between 24.0°C and 29.9°C in summer and 16.5°C to 20.1°C in winter (data provided by the South African Weather Service). The CoJ experiences heat waves relatively frequently, notably in 2014, 2015 and 2016. On 7 January 2016, the maximum temperature of 38°C was the highest recorded temperature for the CoJ, surpassing the November 2015 maximum of 36.5°C. This study was part of a larger project that measured indoor classroom temperatures and entailed schoolchildren recording their heat-health symptoms during class time in selected schools in the CoJ [21]. Between 1 February 2014 and 31 March 2014, household questionnaires with accompanying written informed consent sheets were distributed to parents of children participating in the study on classroom temperature and perceived child heat-health symptoms [21]. Completed questionnaires were returned to the teacher who stored them in a locked cupboard until all questionnaires had been returned for collection and analysis by the researcher.

**Ethics approval and consent to participate:** research ethics clearance was granted by the Tshwane University of Technology Research Ethics Committee (REC2013/11/007) and the Council for Scientific and Industrial Research Ethics Committee (53/2012). Permissions from the Gauteng Department of Education and the National Department of Health, school principals and chairpersons of the school governing bodies were obtained. Parents who completed the questionnaire gave their written consent.

**Questionnaire:** the questionnaire and heat-related questions were adapted from a household questionnaire that was used to examine household vulnerability to extreme heat among slum dwellers in Ahmedabad, India [23]. It comprised six sections: 1) general household information; 2) household socio-economic factors and health status of each household individual; 3) living conditions and dwelling characteristics; 4) availability of community resources; 5) behavioural attributes and social ties; and 6) perceptions, awareness and attitudes on climate change and health. Here, we only report on parts 1 to 3 pertaining to socio-economic and dwelling risk factors in relation to reported health outcomes. The questionnaire contained a hierarchical, multi-level structure: level 1 included the individual data of each household member (i.e. age, sex, marital status, highest education level, occupation, income, heat symptoms, diseases) and level 2 comprised data about the household. Questionnaires were included for analysis only when section 2 (health status of each household individual) had been completed in full for every household member, where a household member was defined as someone who usually eats with the remaining household members. Failure to complete section 3 was also considered an exclusion criterion. We assumed that all individuals living in the same dwelling were exposed to the same household risk factors.

**Statistical analyses:** statistical analyses were performed using STATA version 14.0 [24]. Bivariate logistic regression was conducted to assess associations between 'any heat-health effects' and risk factors [25]. These were (a) existing chronic conditions and (b) age group and gender. Bivariate analysis was used to examine associations between individual reported 'any heat-health effects' and living conditions / dwelling characteristics as risk factors [26]. Crude Odds Ratios (ORs) and their respective 95% confidence intervals (CIs) and p-values were calculated by means of Chi-squared tests and Wald Chi-Square (one-tailed and two-sided) tests. Variables were considered significant at the p ≤ 0.05 level.

## Results

**Individual heat-health effects:** four hundred and eighty household questionnaires were distributed, and the overall parent / caregiver response rate was 45% (n=217). After applying the inclusion criteria, i.e. fully completed questionnaire sections 2 and 3 (see methods section above for full explanation), a total of 136 household questionnaires (28%) were eligible for inclusion, and data for 136 households and 580 individuals were evaluated. Primary household caregivers were asked about heat-health symptoms experienced by household individuals when the weather is hot (Table 1). These heat-health symptoms included sweating, weakness, fatigue and dizziness, and muscle cramps. For doctor-diagnosed chronic health outcomes experienced by household individuals as reported by the primary household caregiver, the highest prevalences were reported for hypertension and stress, followed by skin rash / dermatitis and 'any chronic injuries' (Table 2).

**Table 1:** Heat-health effects experienced by household individuals as reported by the primary caregiver for the question ‘is there any member of your family who experiences the following symptoms / outcomes when it is hot?’

Health outcome	Number of individuals (N=580) (n)	Percentage of total participants (%)
Sweating	208	35.8
Headache and nausea	111	19.1
Weakness, fatigue and dizziness	87	15.0
Muscle cramps	60	10.3
Hot, dry and flushed skin	41	7.0
Shallow breathing	35	6.0
Irregular heart beat	33	5.6
Agitation and confusion	27	4.6
Cold, pale and clammy skin	27	4.6
Increased respiratory rate	27	4.6
Fainting	21	3.6
Weak rapid pulse	15	2.5
Shock	13	2.2
Decreased level of consciousness	12	2.0
Seizures	11	1.8
Any other symptoms	11	1.8
Cardiac arrest	6	1.0

**Table 2:** Chronic health outcomes experienced by household members as reported by the primary caregiver for the question “Has any member of your family ever been told by a doctor that they have any of the following illnesses?”

Chronic health outcome	Number of individuals (N=580) (n)	Percentage of all individuals (%)
Hypertension / High blood pressure	71	12.2
Stress	70	12.0
Skin rashes and / or Dermatitis	37	6.3
Chronic injury (any)	32	5.3
Diabetes	25	4.3
Asthma	25	4.3
Depression	24	4.1
Bronchitis	22	3.7
Arthritis	21	3.6
Overweight and / or Obese	17	2.9
Anxiety	16	2.7
Chronic fatigue	10	1.7
Heart disease	8	1.3
Mental conditions (any)	6	1.0
Cancer	3	0.5
Disability (any)	3	0.5
Epilepsy	2	0.3
Parkinson’s disease	1	0.1
Alzheimer’s disease	0	0.0

**Chronic health outcomes in relation to individual 'any heat-health effects':** using the doctor-diagnosed health outcomes in Table 2, we combined any / all heat-health effects reportedly experienced by an individual to create a new variable 'any heat-health effects' reportedly experienced by an individual and considered chronic health outcomes and socio-demographic variables as potential risk factors.

**Associations between 'any heat-health effects' and risk factors:** age was a significant risk factor for 'any heat-health effects' (Table 3). Individuals older than 60 years were almost two times more likely to experience 'any heat-health effects' compared to individuals in other age groups (OR = 1.81, 95% CI 1.27 - 1.99, p < 0.001). Females were reportedly 1.15 times more likely to suffer from 'any heat-health effects' compared to males although this finding was not statistically significant (OR = 1.15, 95% CI 0.82 - 1.62, p = 0.412). The results of the bivariate analyses showed that hypertension was the only pre-existing chronic health condition that had a statistically significant association with 'any heat-health effects'. Individuals who suffered from hypertension were about two times more likely to experience 'any heat-health effects' (OR = 2.32, 95% CI 1.34 - 4.05, p = 0.003).

**Table 3:** ‘Any heat-health effects’ reportedly experienced by an individual in a household by household living conditions and characteristics of their dwelling

			Odds of an individual in the household reporting ‘any heat-health effects’		
Variable	Number of individuals (N=580) (n)	Percentage of individuals (%)	Crude Odds ratio	95% Confidence Interval	p-value
Housing tenure					
Own*	375	64.7			0.01
Rent	132	22.8	0.59	0.40 - 0.89	
Air conditioning in dwelling					
Yes*	38	6.6			
No	478	82.4	1.02	0.52 - 1.97	0.05
Type of house					
Detached house (single)*	191	32.9			
Mud house	10	1.7	1.48	0.37 - 5.89	0.58
Townhouse	55	9.4	1.30	0.68 - 2.45	0.40
Flat / apartment	110	19.0	0.39	0.24 - 0.63	0.00
RDP-type house	92	15.9	2.01	1.15 - 3.52	0.01
Mobile house	22	3.7	0.36	0.14 - 0.90	0.03
Shack	32	5.5	0.60	0.20 - 0.93	0.03
Type of roofing					
Tiles*	143	40.5			
Thatch	7	1.21	0.12	0.01 - 0.90	0.04
Asphalt shingles	9	1.6	0.18	0.04 - 0.90	0.04
Asbestos sheets	76	13.1	1.04	0.61 - 1.77	0.89
Corrugated iron sheets	155	26.7	0.74	0.49 -1.12	0.15
Electricity in the house					
Yes*	533	92.0			
No	43	7.4	1.20	0.63 - 2.26	0.58
Separate room inside house for cooking					
Yes*	533	91.9			
No	43	7.4	0.72	0.47 - 1.12	0.15
Fuel used for cooking					
Electricity*	547	94.3			
Gas	9	1.6	2.72	0.56 - 13.19	0.21
Paraffin	10	1.7	0.78	0.22 -2.71	0.69
Combination of fuels	3	0.5	1.00	-	-
Firewood	9	1.6	0.22	0.04 -1.08	0.06
Coal	2	0.3	1.00	-	-
Fuel used for lighting					
Electricity*	543	93.6			
Liquid Petroleum Gas	0	0.0	-	-	-
Paraffin	21	3.6	1.56	0.62 - 3.92	0.35
Combination of fuels	0	0.0	-	-	-
Candles	9	1.6	0.22	0.05 -1.08	0.06
Piped water in the house					
Yes*	481	82.9			
No	70	12.0	0.90	0.55 -1.50	0.70
Toilet inside the house/yard					
Yes*	551	95.0			
No	19	3.2	1.14	0.46 - 2.85	0.78

Note: *indicates the reference category

**Household living and dwelling characteristics in relation to individual 'any heat-health effects':** most families lived in detached houses, followed by flats or apartments and government-sponsored (a South African Reconstruction and Development Programme (RDP) detached house) houses (Table 3). Most dwellings had a tiled roof followed by houses with a corrugated iron roof. Four-fifths of dwellings had a dedicated, separate kitchen for cooking. Households mainly had electricity as their primary energy carrier for cooking and lighting followed by paraffin. Less than 2% of households used solid fuel, i.e. coal or wood, for cooking. Most households had in-house electricity and piped water. Almost all dwellings had a toilet inside the house or yard (asked together as one question). Less than 10% of individuals lived in households with air-conditioners. Living in a government-sponsored house was associated with an increase in 'any heat-health effects' (Table 3). Individuals living in dwellings with roofs made from asbestos sheets had the highest odds of reporting heat-health effects compared with people living in dwellings with other roof types, although this finding was not statistically significant.

## Discussion

We aimed to determine risk factors associated with heat-health effects experienced during hot weather by individuals living in an African city. People living in poverty as well as those communities living in urban environments are considered as being most vulnerable to the risks associated with increasing temperatures in Africa [27]. Hence, we focused on an urban African setting where special attention is needed to prevent adverse health impacts in a changing climate. We found that, for non-modifiable risk factors, individuals over the age of 60 years were reportedly most likely to experience 'any heat-health effects'. Several studies have shown that people above 60 years are at increased risk of heat-related illness because of physiological impairments in the regulation of core body temperature in hot conditions [17, 28, 29]. Females were reported to be more likely to experience any heat-health symptoms than men although this finding was not statistically significant. This was also found during the Euro HEAT project that considered the impact of heat waves on mortality in nine European cities [30]. Over the summers of 1990 to 2004 and in the year 2003, separately, the highest effect from heat on mortality was observed among women aged 75 to 84 years. Women may be more vulnerable to the effects of heat in African urban settings where they spend considerable time indoors caring for children, the elderly and looking after their dwellings. Regarding pre-existing diseases in relation to heat-health effects, our findings agreed with previous studies [28, 31, 32] where hypertension was a statistically significant risk factor for an individual to experience 'any heat-health effects'. Adaptation strategies need to be implemented to prevent heat-related morbidity and may include cooling the room in which the person is located, and possibly also changing medical treatment doses during hot weather [32]. African cities are renowned for having a mix of different housing types in terms of nature, design, multi-use (e.g. cottage industries) [33] and building materials, among other factors. Here, we found that living in RDP housing was associated with an increase in reported experience of 'any heat-health effects' compared to living in other housing types, e.g. flat / apartment and informal dwelling or shack. RDP homes built by contractors are often very small and of poor quality [34]. In a recent study, also in the CoJ, low-cost RDP houses had the greatest variation in indoor temperature and experienced indoor temperatures 4 to 5°C higher compared to ambient outdoor temperatures [35]. These types of houses are typically built from bricks (either cement or a combination of sand and cement) with high thermal conductivity and an exterior wall of single bricks no more than 15 cm thick. They also do not usually have ceilings or thermal insulation in ceilings [36] thereby reducing their thermal comfort.

Despite not being statistically significant, the strongest effect for a modifiable risk factor in relation to 'any heat-health effects' was found for living in a dwelling with asbestos sheet roofing. Asbestos roofs can increase indoor temperatures due to an intense transfer of heat into the internal environment during hot weather [37, 38]. In Sri Lanka, excessive heat transferred through the asbestos roof was one of the main causes of thermal discomfort in warm, humid climate conditions [39]. There, a cement-asbestos fibre sheeting roof compared to clay tile roofing resulted in warmer indoor temperatures. While asbestos roof sheeting is banned in South Africa [40], it was widely installed in low-cost housing in the past and remains in place in some dwellings throughout the country. In a survey of 1 488 households in Soweto in the CoJ, 52% of dwellings had a corrugated asbestos roof [41]. In addition to the heat-health induced temperature effects of asbestos leading to warmer indoor environments, asbestos fibres when disturbed become airborne and are associated with asbestosis and various cancers [42]. Rehabilitation of asbestos roofing therefore needs to be addressed professionally and with care for residents' health. The study was constrained in design by using a sample made during another study and by asking the parent / primary caregiver to provide information about the house as well as all demographic and health information for every individual living in the house. We also assumed that the primary household caregiver understood the terminology used for heat-health effects and chronic health outcomes. We did not collect all potential confounders, nor did we detail severity of heat-health effects contained in the questionnaire. We also did not measure temperature inside any of the respondents' houses, nor did we specify hot weather events / hot weather referred to in the questionnaire when we asked respondents to explain which symptoms they themselves as well as members of their family experienced during such hot weather. We also asked one question about whether the toilet was inside the house or in the yard - this question should have asked about 'inside' and 'in yard' toilets separately given the public health consequences of sanitation locations. The small sample and poor response, likely due to the request for detailed information about each individual living in the household and a self-completed questionnaire format from the primary caregiver, resulted in relatively low disease prevalence and subsequent wide CIs despite statistically significant odd ratios, hence these risk factors should be tested in a larger sample.

## Conclusion

We have highlighted specific risk factors that should be considered when trying to reduce adverse heat-health effects among vulnerable groups living in urban African settings. We have identified ways in which individuals and policy makers can act. Specifically, due to their vulnerability to heat, people with pre-existing diseases (especially hypertension) and the elderly should be targeted for heat-health information on ways to cope during hot weather. Our findings provide information that can inform the development of community heat and health adaptation awareness campaigns and prevention of adverse heat-related health impacts in African urban settings.

### What is known about this topic

- Climate change is occurring and causing temperatures to increase especially in southern Africa;
- Heat-related health impacts are well understood in individuals working in occupational settings such as in underground mines. Less has been done in community settings and even less in the African context.

### What this study adds

- We determined that risk factors associated with heat-health effects experienced during hot weather by individuals living in an African city were age, gender and housing type;
- Older people, women who spend much time in the home and people living in a home with asbestos roofing were all indicative of groups that need attention in the future to ensure healthy homes, and good health and wellbeing for all individuals especially during periods of high temperature;
- These findings build on the body of evidence needed in Africa to provide for evidence-informed policy advice towards heat-health adaptation plans.

## Competing interests

The authors declare no competing interests.
